# Scope and Prospects of Social Media for Patient Education and Engagement in Medical Practice

**DOI:** 10.7759/cureus.106411

**Published:** 2026-04-03

**Authors:** C Kishore Kumar, R Premaraja, Sargunan Arulraja, Shiji M Kunjappan, Thangadurai Maheswaran, Sivanesan Sathiya Narayana Murthy, Kanmani Raju, M Saranya

**Affiliations:** 1 Physiology, Takshashila Medical College, Takshashila University, Ongur, IND; 2 Physiology, Melmaruvathur Adhiparasakthi Institute of Medical Sciences and Research, Melmaruvathur, IND; 3 Pharmacology, Swamy Vivekanandha Medical College Hospital and Research Institute, Namakkal, IND; 4 Orthodontics, PSM College of Dental Sciences and Research, Thrissur, IND; 5 Oral and Maxillofacial Pathology, Adhiparasakthi Dental College and Hospital, Melmaruvathur, IND; 6 Anatomy, Shri Sathya Sai Medical College and Research Institute, Sri Balaji Vidyapeeth (Deemed to be University), Puducherry, IND; 7 Oral Medicine and Radiology, Chettinad Dental College and Research Institute, The Tamil Nadu Dr. MGR Medical University, Chennai, IND; 8 Oral Pathology and Microbiology, Vivekanandha Dental College for Women, Namakkal, IND

**Keywords:** artificial intelligence, facebook, health communication, medical practice, patient education, social media, twitter, whatsapp, youtube

## Abstract

Social media has fundamentally altered the landscape of health communication, creating new opportunities for patient education, peer support, and clinical engagement in medical practice. Platforms such as Facebook, YouTube, X (formerly Twitter), and Instagram now serve as primary conduits for health information, enabling patients with acute and chronic conditions to seek guidance, compare clinical experiences, and build supportive communities. Literature shows that well-implemented social media strategies can improve self-management behavior, medication adherence, and disease awareness across diverse patient populations. However, these gains are counterbalanced by substantive risks, including the proliferation of misinformation amplified by automated bots, ethical dilemmas surrounding professional boundaries and patient confidentiality, and the uneven quality of user-generated health content. Artificial intelligence (AI) is increasingly being integrated with social media channels to provide personalized, scalable health information and chatbot-assisted patient education. This narrative review examines current literature to delineate the scope and prospects of social media for patient education and engagement and to propose best practices for clinicians navigating this rapidly evolving digital environment.

## Introduction and background

The emergence of social media as a dominant channel of global communication has substantially reshaped the patient-clinician dynamic. In the past, people shared information mainly in consultation rooms. Now, millions use platforms like Facebook, X (formerly Twitter), Instagram, and YouTube to find, share, and check health information [1,2]. This transition mirrors a broader shift toward patient-centered care, in which patients increasingly expect transparency, accessibility, and dialogue rather than one-directional instruction from healthcare providers.

The practical implications of this shift are considerable. Surveys across diverse specialties confirm that a substantial proportion of patients research their diagnoses and treatment options online before presenting to their provider, while growing numbers consult peer communities to fill perceived gaps in institutional health communication [3,4]. Meanwhile, healthcare professionals have begun to leverage social platforms for professional development, academic dissemination, and patient outreach, even as the boundaries of appropriate online clinical conduct remain incompletely defined [5,6].

Despite these developments, the evidence base for optimal social media use in clinical medicine remains fragmented. Most published data originate from analyses specific to particular specialties, such as orthopedics, dermatology, pathology, and oncology, rather than from cross-disciplinary, multi-specialty frameworks [7,8]. The dual potential of social media to both advance and undermine patient health is particularly salient in the context of information integrity: the same platforms that enable life-changing peer connections also serve as conduits for dangerous health misinformation [9,10].

This narrative review examines the current literature on the scope and prospects for patient education and engagement in modern medical practice.

## Review

Digital shift in health communication

The penetration of smartphones and affordable mobile internet has accelerated a fundamental transformation in how patients access health information. Social media platforms now function as dynamic, interactive health ecosystems in which patients post questions, read peer narratives, and encounter clinical content from both verified professionals and unvetted lay users [1,2]. This digital health transition predates the COVID-19 pandemic but was substantially amplified by it, as lockdowns eliminated routine in-person consultations and drove unprecedented traffic to online health communities [11]. Surveys of primary care populations indicate that a majority of patients in high-income countries actively use the Internet for health research, with a significant and growing proportion turning specifically to social media rather than professional health portals [1].

This shift has important implications for medical practice. The traditional information asymmetry between clinicians and patients is eroding; patients arrive at consultations with preformed opinions shaped by online communities, influencer content, and peer testimonials [4]. Rather than viewing this as a threat to clinical authority, evidence supports reframing the clinician's role as a trusted curator of quality information within the digital ecosystem [7]. Integrating an awareness of patients' digital information-seeking behavior into the clinical workflow represents a necessary adaptation, not an optional supplement, to contemporary medical practice [1,7].

Platform characteristics

Different social media platforms offer distinct functional architectures that shape their utility and limitations in healthcare contexts. X facilitates the rapid dissemination of brief, hashtagged content and has proven particularly effective for professional medical networking, live conference engagement, and the sharing of educational case material, as demonstrated extensively within hematopathology [8]. Facebook, with its closed-group functionality, enables more private, condition-specific communities where patients exchange treatment experiences and manage ongoing illness narratives. YouTube accommodates longer-form video content suited to procedural demonstrations and patient education sequences, whereas Instagram's visual format supports campaigns in dermatology, surgical procedures, and public health messaging [7,2].

The choice of platform influences the nature and quality of information encountered. A systematic review of orthopedic surgery found that Instagram is the most widely adopted platform among training programs, followed by X and Facebook, with each supporting different engagement objectives [7]. Within pediatric subspecialties, social media has been effectively deployed to disseminate clinical guidelines and engage patients' families directly [12]. The absence of formal platform selection guidance in most institutional policies represents a gap that professional bodies are only beginning to address [8,2].

Benefits for patient education

Social media platforms offer several substantive advantages for patient education relative to traditional communication modalities. The promptness, accessibility, and multimedia flexibility of platforms such as Facebook, YouTube, and X enable clinicians to reach diverse patient populations outside formal care settings [1,3]. Content can be tailored to specific conditions, literacy levels, and cultural contexts, whereas interactive features like comment threads, polls, and direct messaging foster bidirectional engagement that static leaflets and websites cannot replicate.

Oncology provides one of the most studied domains. In breast cancer care, social media has served as a vehicle for information dissemination about diagnosis, treatment options, survivorship, and reconstruction, reaching patients at geographically and socioeconomically diverse locations [3]. Patients report that access to online communities and clinician-authored content significantly reduces decisional uncertainty and enhances consultation preparation. Similarly, a survey-based study among families of children with microtia found that 90.5% of respondents believed that their surgical specialists should maintain an active social media presence to facilitate education and treatment exploration, while only 46% of surgeons maintained an active account [4]. These data collectively underscore that patients perceive clinician-led social media content as a legitimate educational resource and that its absence may leave patients reliant on less reliable sources [1,8].

Role in chronic disease management (CDM)

CDM represents one of the most clinically productive applications of social media in healthcare, given the ongoing self-management demands of conditions, such as diabetes, chronic obstructive pulmonary disease (COPD), and renal failure. A randomized controlled trial demonstrated that a social media-based pulmonary rehabilitation program delivered via WeChat significantly improved self-efficacy, symptom burden, and quality of life in patients with COPD, performing comparably to face-to-face rehabilitation over six months [13]. These findings illustrate the potential of social messaging platforms to extend the therapeutic reach of CDM programs beyond the clinic walls.

In diabetes, social media communities have enabled patients to share real-time glycemic data, dietary strategies, and device experiences [14,15]. A landmark survey of an international social media community for individuals with type 1 diabetes mellitus following a very low-carbohydrate diet reported exceptional glycemic control and low rates of adverse events, suggesting that peer-mediated information exchange can positively influence clinical outcomes [14]. The American Association of Diabetes Educators has formally recognized peer support communities as integral to diabetes self-management education, encouraging structured collaboration between clinical teams and online patient networks [15]. Artificial intelligence (AI) integration further augments chronic disease support by enabling automated symptom monitoring and personalized patient feedback [16].

Online peer support and mental health

Online peer support has emerged as a significant adjunct to formal mental health care, particularly for individuals with serious mental illness (SMI) who face barriers to in-person treatment. A conceptual model proposed by Naslund et al. demonstrated that online peer connections among people with SMI may promote consumer activation, challenge stigma, and facilitate access to interventions for mental and physical well-being [17]. Platforms such as Facebook and X enable individuals to share coping strategies, personal recovery narratives, and practical guidance on navigating mental health services, fostering a sense of group belonging that can buffer social isolation [17].

In adolescent populations, digital media use is particularly relevant for peer support, mental health disclosure, and identity development [18]. Online platforms provide adolescents from historically marginalized groups with accessible spaces for peer connection and affirmation. However, the same channels carry risk: cyberbullying, social comparison, and exposure to harmful content have been associated with increased psychological distress, and evidence points to a U-shaped association between internet use and depression [18]. For patients managing diabetes, the psychosocial dimension, including burnout and distress, is increasingly addressed within online peer communities, where shared lived experience complements formal clinical education [15]. Critically appraising the quality and safety of peer interactions is therefore essential before recommending specific communities to vulnerable patient populations [17,18].

Professional ethics and boundary management

The integration of social media into clinical practice introduces complex ethical challenges that extend beyond conventional professional standards. Chief among these are the maintenance of therapeutic boundaries, protection of patient confidentiality, and management of dual online identities, personal and professional. Sabin and Harland identified four major ethical challenges for clinicians using digital technologies with patients: boundary management, privacy and confidentiality, realistic expectations about digital communications, and upholding professional ideals [19]. Traditional ethical frameworks remain applicable in digital contexts; however, their application requires context-specific adaptation for online environments in which information persistence and searchability fundamentally alter risk profiles.

In dermatology, clinicians who operate as online influencers face documented risks, including breaches of patient confidentiality, financial conflicts of interest, and inadvertent dissemination of inaccurate information [5]. Across specialties, the sharing of patient-identifiable data on social media, even with ostensibly educational intent, requires explicit consent and careful anonymization, obligations formalized by legislation such as the General Data Protection Regulation in European jurisdictions [6]. Orthopaedic and pediatric surgery literature confirms that, despite the growing presence of clinicians on social platforms, formal training in professional online conduct remains inconsistently implemented [12,2]. Patients surveyed across multiple studies report searching for personal and professional information about their clinicians online, a reality that demands proactive attention to digital identity management [19,5].

Health misinformation

The proliferation of health misinformation represents perhaps the most consequential risk associated with social media in medical practice. A systematic review of reviews found that the proportion of health-related misinformation on social platforms ranges from 0.2% to 28.8%, with X, Facebook, YouTube, and Instagram identified as the principal vectors of rapid and far-reaching false health content [9]. The COVID-19 pandemic crystallized these dynamics, generating a global "infodemic" in which inaccurate content about transmission, treatment, and vaccines spread faster than verified public health guidance [11,10].

Automated bot accounts play a disproportionate role in amplifying misinformation. A secondary analysis of known bot datasets during the pandemic found that up to 66% of active bots were generating COVID-19 content and exploiting human tendencies to share emotionally resonant information regardless of accuracy [11]. The public health consequences are real and measurable: a systematic review links infodemic exposure to psychological distress, vaccine hesitancy, misallocation of health resources, and increased incidence of fear- and panic-driven behaviors [10,9]. Countermeasures identified through expert conference analysis include rebuilding public trust in science, redesigning algorithmic recommendation systems to limit the spread of misinformation, fostering interorganizational partnerships, and investing in digital health literacy programs [20,21]. Social media literacy, the capacity to critically evaluate online health information, has been demonstrated as a protective factor against infodemic harm, with educational interventions improving the acceptance of evidence-based health behaviors across diverse populations [21]. The integration of AI-driven search tools has introduced additional risks: generative search interfaces have been shown to direct users toward illegal or unverified health resources, including controlled-substance vendors, underscoring the inadequacy of algorithmic curation alone [22].

Role of AI

AI is rapidly reshaping the interface between social media and health communication, introducing capabilities that extend beyond what human-only platforms can deliver. In diabetes care, AI algorithms integrated with digital platforms support predictive risk modelling, continuous remote monitoring, clinical decision support, and personalized self-management coaching, augmenting the engagement functions of peer communities [16]. Large language model-based chatbots have demonstrated particular promise in responding to patient queries on social media forums. A cross-sectional study comparing ChatGPT and verified physician responses to patient questions posted on a public online forum found that evaluators rated AI-generated responses as significantly superior in terms of informational quality and empathetic tone, with AI responses rated as good or very good quality 3.6 times more often and rated as empathetic or very empathetic nearly ten times as frequently [23].

Clinical applications of AI chatbots in direct patient care have extended into nursing and specialist settings. The implementation of a LINE-based chatbot for patients with peritoneal dialysis yielded patient satisfaction rates exceeding 91%, reduced infection rates at the catheter exit site, and outcomes comparable to those of face-to-face education programs, underscoring the scalability of AI-assisted education in complex chronic care [24]. Notwithstanding these advances, the integration of generative AI into consumer-facing search and social media platforms has produced safety vulnerabilities, including the inadvertent promotion of illegal online pharmacies and the provision of misguided drug information to patients seeking prescription medications [22]. Rigorous oversight frameworks and clinician-guided AI deployment remain essential prerequisites for safe implementation [23,16].

Best practices for clinicians

Evidence supports a structured approach to social media engagement for clinicians seeking to harness its educational and communicative potential while managing associated risks. Foremost among best practices is the establishment of a clearly delineated professional online identity separate from personal social media activity, which mitigates boundary violations and maintains the dignity of the therapeutic relationship [1,5]. Clinicians should obtain and document explicit patient consent before sharing any case material online, adhere to institutional social media policies, and familiarize themselves with applicable data protection legislation [6].

Active participation in specialty-specific hashtag communities, hosting of live question-and-answer sessions, and curation of evidence-based content represent strategies demonstrated to enhance both reach and professional reputation [12,3]. Clinicians are advised to regularly audit their own digital presence, correct misinformation they encounter, and refer patients to vetted health information resources rather than unmoderated peer forums when appropriate [5]. Patient education content should prioritize accessibility, cultural sensitivity, and medical accuracy and should be reviewed periodically in light of evolving evidence. Institutions are responsible for supporting formal training in digital professionalism, a domain currently underrepresented in most postgraduate medical curricula [1,7]. A collaborative model, in which clinicians, professional societies, platform developers, and patient advocacy groups co-develop and enforce community standards, represents the most sustainable architecture for high-quality digital health communication.

## Conclusions

Social media has become an indispensable and increasingly integral element of health communication within contemporary medical practice. Its ability to facilitate patient education, enhance CDM, cultivate therapeutic peer communities, and enable clinicians to disseminate evidence-based information on a large scale represents a significant advancement in patient-centered care. The evidence reviewed herein demonstrates measurable benefits across a wide array of conditions and specialties, ranging from improved glycemic control in diabetes communities to enhanced survivorship support in breast cancer, from reduced infection rates through AI chatbot-assisted education to substantial mental health improvements via peer networking. However, it is important to recognize that the evidence base remains heterogeneous in quality, with variability in study design, population characteristics, and outcome measures. Consequently, many observed benefits are context-dependent and not yet standardized or consistently generalizable across diverse clinical settings. Simultaneously, the risks associated with misinformation, ethical boundary violations, privacy breaches, and the algorithmic amplification of harmful content are well-documented and cannot be addressed through disengagement alone.

For clinicians, the primary implication is that passive withdrawal from social media is no longer a feasible professional strategy. Patients are already active on these platforms, seeking information, community, and validation; the pertinent question is whether clinicians will engage in shaping that informational environment. The actionable priorities emerging from this review include investment in formal digital professionalism training within medical education, adoption of specialty society guidelines on online conduct, proactive correction of health misinformation, and collaboration with platform developers and public health authorities to enhance algorithmic accountability. By adopting a thoughtful, evidence-informed approach to social media engagement, clinicians can transform a reactive challenge into a proactive tool for improving patient health outcomes at a population scale.
